# [Corrigendum] Human papillomavirus E6‑regulated microRNA‑20b promotes invasion in cervical cancer by targeting tissue inhibitor of metalloproteinase 2

**DOI:** 10.3892/mmr.2023.12988

**Published:** 2023-03-29

**Authors:** Yuan Cheng, Li Geng, Lijun Zhao, Peng Zuo, Jianliu Wang

Mol Med Rep 16: 5464–5470, 2017; DOI: 10.3892/mmr.2017.7231

Subsequently to the publication of this paper, an interested reader drew to the authors’ attention that, in the wound-healing assay shown in Fig. 2C on p. 5467, the data panel for the ‘Anti-NC / 24 h’ experiment appeared to be the same as that shown for the ‘miR-NC / 0 h’ data panel, albeit the image had been turned through 180°.

After having re-examined their original data, the authors have realized that this figure was inadvertently assembled incorrectly. The corrected version of Fig. 2, now showing the correct data for the ‘Anti-NC / 24 h’ panel in Fig. 2B, is shown on the next page. Note that this error did not significantly affect the results or the conclusions reported in this paper, and all the authors agree with the publication of this Corrigendum. Furthermore, the authors apologize to the readership for any inconvenience caused.

## Figures and Tables

**Figure 2.**
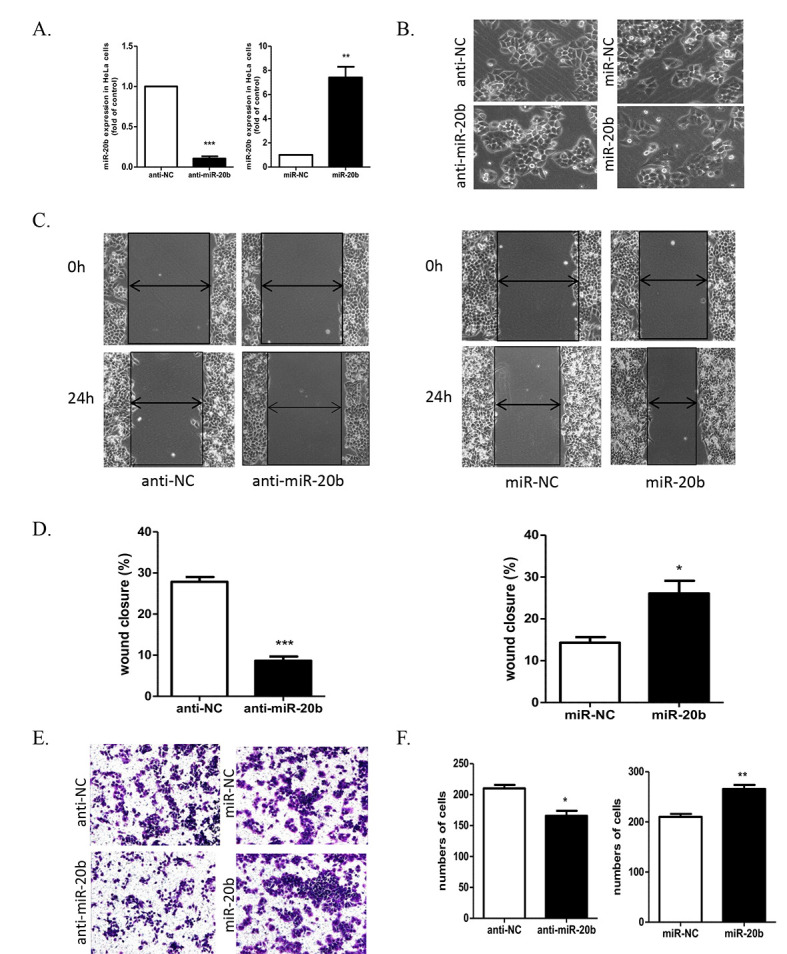
miR-20b induced morphologic changes of HeLa cells and promoted their migration and invasion. (A) Cells were transfected with miR-20b inhibitor (anti-miR-20b) at 100 nm and miR-20b mimics (miR-20b) at 50 nm. Data are mean ± SEM (n=3). **P<0.01, ***P<0.001 vs. control. (B) Microscopy of cell migration before and after miR-20b inhibition or overexpression. (C and D) Scratch assay of cell migration and (E and F) Transwell invasion assay of invasion with miR-20b inhibition and overexpression. Data are mean ± SEM (n=3). *P<0.05, **P<0.01, ***P<0.001 vs. control.

